# Open surgery retrieval of a missing guidewire causing cerebral infarctions after cerebral angiography: a case report

**DOI:** 10.1186/s13019-021-01531-w

**Published:** 2021-05-29

**Authors:** Chaodi Luo, Jing Li, Yang Yan, Dan Han

**Affiliations:** 1 Department of Cardiology, the First Affiliated Hospital of Xi’an Jiaotong University, Xi’an, PR China; 2 Department of Cardiovascular Surgery, the First Affiliated Hospital of Xi’an Jiaotong University, 277 Yanta West Road, Xi’an, Shaanxi 710061 PR China

**Keywords:** Intra-aortic foreign body, Guidewire, Sternotomy, Cerebral infarctions

## Abstract

**Background:**

Intra-aortic foreign body (IAFB) is uncommon, which is usually caused by a rupture of the catheter or guidewire. IAFB can cause catastrophic complications, including arrhythmia, embolization of guidewire fragments, intravascular clipping of the guidewire and vascular perforation. However, there are still no guidelines on removal and management of IAFB. Here, we present a rare case of fractured cerebral angiographic guidewires in the aorta that resulted in multiple cerebral infarctions.

**Case presentation:**

A 50-year-old man experienced new cerebral infarction after cerebral angiography. Computed tomography and echocardiography demonstrated foreign bodies in his ascending aorta and aortic arch. Open surgery was successfully performed to retrieve the guidewires. The postoperation and follow-up was uneventful.

**Conclusion:**

It is very important for interventional radiologists to check the catheter and guidewire after operation and perform ultrasound or radiograph to prevent IAFB. Additionally, the effective management of IAFB requires the early detection and the selection of appropriate treatment options, as well as long-time follow up.

**Supplementary Information:**

The online version contains supplementary material available at 10.1186/s13019-021-01531-w.

## Introduction

With the expanding application of catheters and the implementation of invasive procedures, interventional radiologists are increasingly faced with foreign body retention or iatrogenic placement of equipment, such as fractured guidewires or catheters. Under normal conditions, the guidewire will be removed after the catheter is inserted into the vessel, which is generally safe and simple. However, infrequent incident can occur when resistance is encountered during removal, the guidewires are usually cut at the skin surface and retained in the vessel, which may cause catastrophic complications, including arrhythmia, embolization of guidewire fragments, intravascular clipping of the guidewire and vascular perforation [1–4].

Intra-aortic foreign body (IAFB) is uncommon, which is usually caused by a rupture of the catheter or guidewire during central venous catheterization. Due to the uncertainty and high risk of IAFB, it is recommended to remove them as soon as possible. There are still no guidelines on the removal of foreign bodies, and the management mainly depends on the specific characteristics of the case and the experience of the surgeon. Here, we present a rare case of fractured cerebral angiographic guidewires in the aorta that resulted in multiple cerebral infarctions.

## Case presentation

A 50-year-old man was admitted to our cardiovascular surgery department with an 8-month history of dizziness and a discovery of IAFB for 4 months. He had a sudden dizziness 8 months ago and the computed tomography (CT) indicated cerebral stem infarction. Cerebral angiography was performed at the local hospital, and he was treated with aspirin and atorvastatin calcium. About 4 months ago, the symptom of dizziness reappeared with limited movement of left upper limb. CT revealed new multiple cerebral infarctions and IAFB. The patient was therefore prescribed with rivaroxaban 10 mg/d for 6 months. In order to retrieve the IAFB, the patient was transferred to our hospital.

**Fig. 1:**
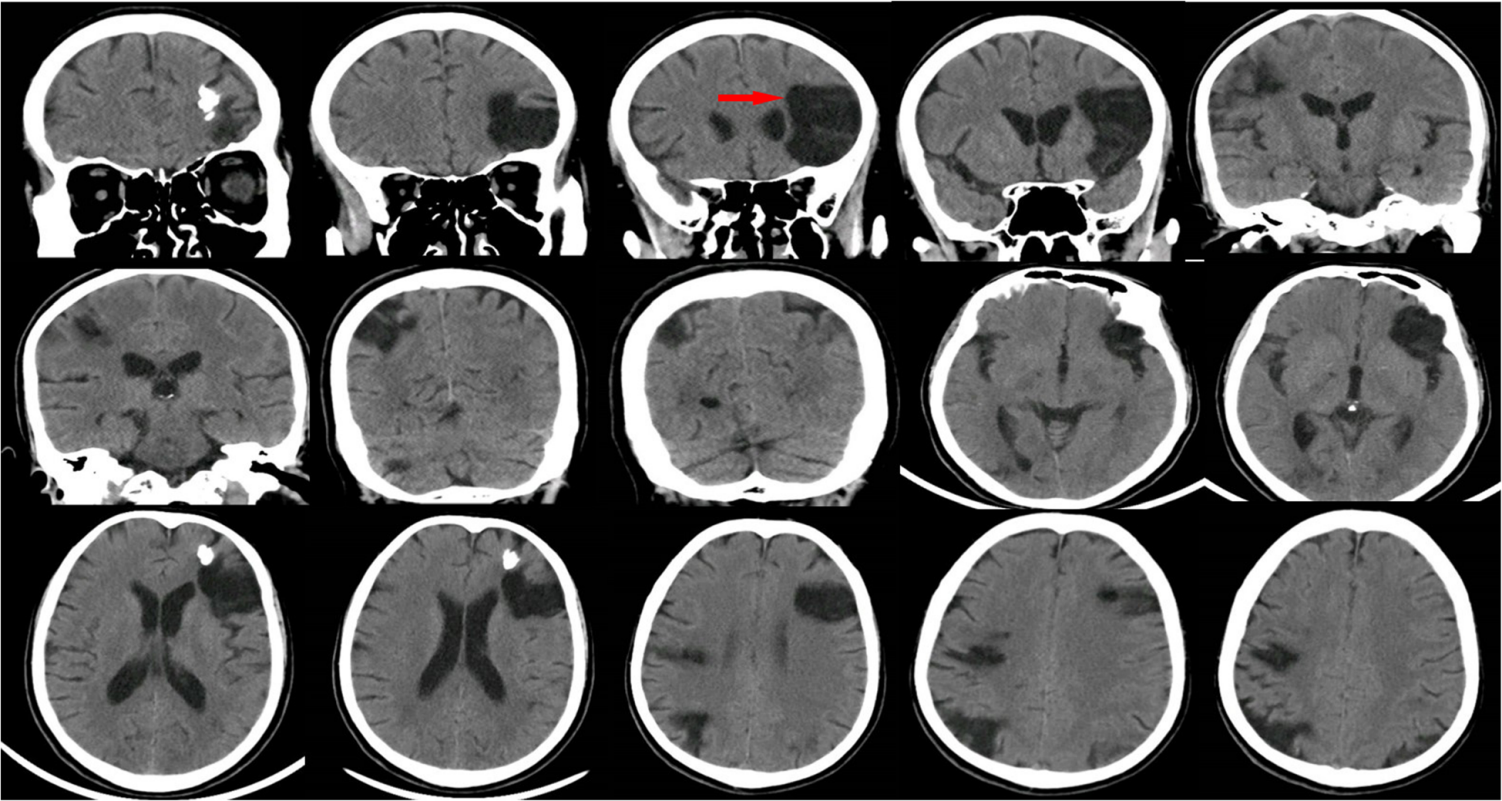
Computed tomography (CT) indicated cerebral infarctions (red arrow)

**Fig. 2:**
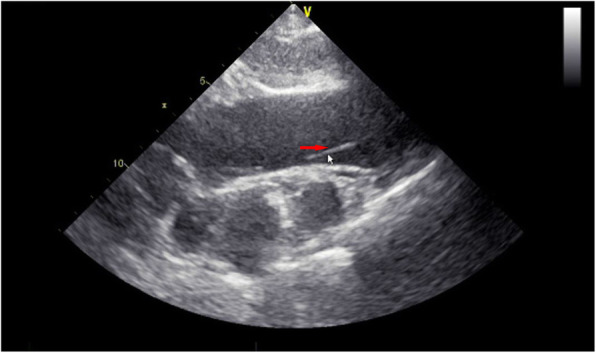
Echocardiography revealed foreign body with strong echogenicity in the ascending aorta and aortic arch (red arrow)

**Fig. 3:**
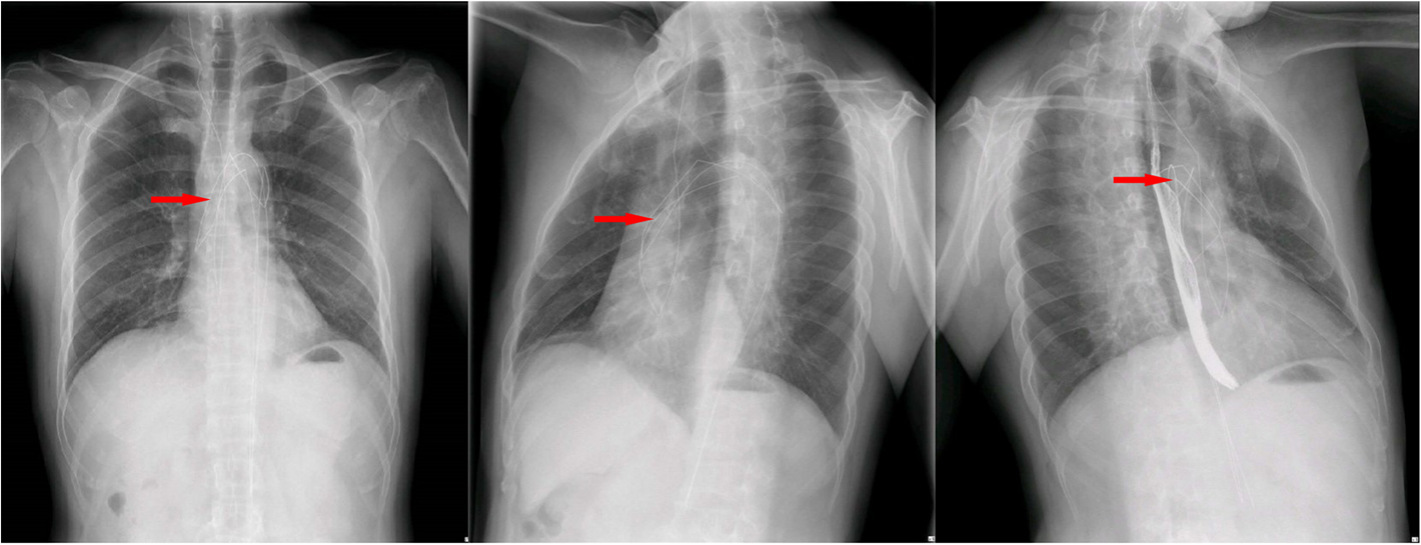
Cardiac three-dimension radiograph showed a linear high-density shadow in the aorta, which partly wrapped around the aortic arch, and one end entered the right common carotid artery (red arrow)

The physical examination of the patient showed no obvious abnormalities. The neurological examination showed the left upper limb muscle strength score was level 2. Brain CT scan revealed multiple softening foci appeared in left frontal lobe, right parietal lobe and right cerebellar hemisphere (Fig. 1). Echocardiography demonstrated strong echo foreign bodies in ascending aorta and aortic arch (Fig. 2, Supplemental video 1). A further three-dimension X-ray images revealed a linear, high-density shadow within the aorta, part of which was wrapped around the aortic arch, with one end entering the right common carotid artery and the other end located in the thoracic and abdominal aorta (Fig. 3). He was diagnosed as IAFB and referred for emergent open surgery.

**Fig. 4:**
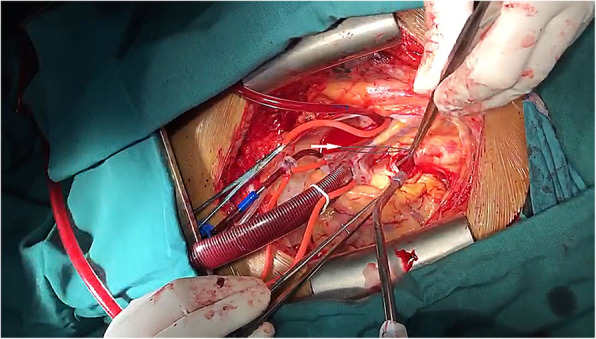
Intraoperative picture showed the operative procedure (the white arrow presenting the guidewire)

**Fig. 5:**
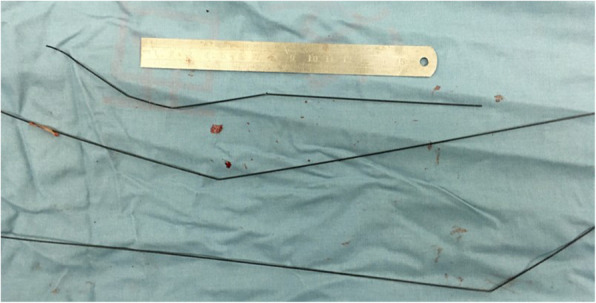
The removed guidewires

**Fig. 6:**
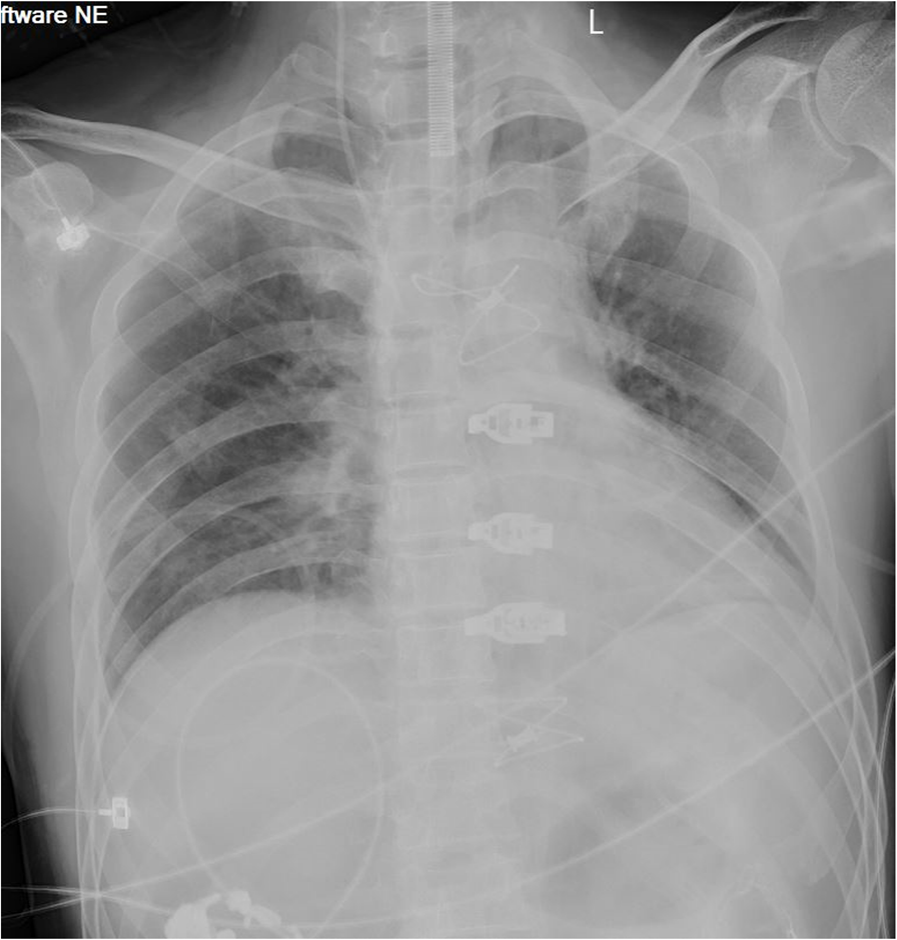
Postoperative radiograph revealed no residual high-density shadows

The patient was placed in the supine position, sternotomy was performed with a mid-sternal incision and fully exposed the heart, ascending aorta and aortic arch. After systemic heparinization, an arterial perfusion tube (18Fr) was inserted via the right subclavian artery and a venous bipolar drainage tube (32/34Fr) was inserted into the right atrium to establish extracorporeal circulation. At the same time, a left heart drain was left in place via the right superior pulmonary vein, and a coronary retrograde perfusion tube was inserted through the right atrium to retrogradely perfuse cardiac arrest fluid (DelNido) through the coronary sinus. After the body temperature dropped to 28 °C, paused the extracorporeal circulation, and the right subclavian artery was selected for selective unilateral cerebral perfusion (10 ml/kg/min). The root of the ascending aorta was incised, three segments of the guidewire could be seen in it, part of which was adhered to the proliferative aortic intima. The proliferative adhesion part was peeled off and the guidewire was removed. Further exploration of the descending part of the aortic arch discovered another section of the guidewire, which was carefully retrieved (Fig. 4, Supplemental Video 2). All of the guidewires were successfully removed from the aorta (Fig. 5), and the post-operative radiograph revealed no residual high-density shadows (Fig. 6). Finally, thorough irrigation with physiological saline solution, the injured aorta was repaired with 3–0 prolene suture and spacer. The chest was closed and a drainage tube was placed in the mediastinum. The patient recovered well without complications, and he had no new cerebral infarction during the follow-up periods.

## Discussion

Digital subtraction angiography (DSA) is nowadays a gold standard for the detection of intracranial artery stenosis, and is gaining popularity because of its safety and minimally invasive advantages [5]. Nevertheless, infrequent but catastrophic complications have been reported, including fragments of the catheter or guidewire forming intravascular foreign bodies, which sometimes lead to cardiac arrhythmia and thromboembolism [6]. Few studies have described the angiographic guidewire completely falls off and enters artery with secondary thrombosis. It is probably because this type of injury is really rare, the patient has not received timely treatment and it is also difficult to be removed through surgery.

Once IAFB is diagnosed, it should be removed as soon as possible to prevent potentially fatal complications, including pulmonary, cerebral, or peripheral arterial embolism secondary to thrombosis, sepsis, malignant arrhythmia, endocarditis, ventricular perforation, or even heart tamponade [7]. In our case, the patient underwent cerebrovascular angiography and the guidewire fell off in the aorta unfortunately, which formed thrombus and caused new cerebral infarctions 4 months later. After retrieving the guidewire, the patient lived freely with no new cerebral infarction.

Due to the rarity of intravascular foreign bodies, the treatment is still challenging. Kashif et al. had described retrieval of a missing guidewire from the left arm under fluoroscopic guidance through a percutaneous procedure [8]. This is also emphasized by other reports that percutaneous minimally invasive surgery is commonly used to remove intravascular foreign bodies, including broken catheters, guidewires, dislocated stents, intraluminal filters and occluders. However, such method is not suitable for our patient. First, the guidewire extended from the internal carotid artery to the descending aorta, and was folded into three sections within the descending aorta. Second, the guidewire remained in aorta for 8 months, and formed adhesion with the vascular intima. Compared to intravascular retrieval techniques, open retrieval is more effective in stents, vertebroplasty cement, inferior vena cava filters and guidewires. The choice of opening or intravascular retrieval depends on the position, location, degree of organ involvement, and the type and shape of foreign bodies in the vessels. Therefore, in our case, the use of percutaneous minimally invasive surgery will have great difficulties and risks, which may easily cause artery rupture, but open retrieval could better decrease the risk of artery scratch or even rupture to reduce mortality.

## Conclusion

With the rapid development of invasive procedures, IAFB is becoming more and more common. It is very important for interventional radiologists to check the catheter and guidewire after operation and perform ultrasound or radiograph to prevent such complications. Additionally, the effective management of IAFB requires the early detection and the selection of appropriate treatment options, as well as long-time follow up, which are critical to the patient’s prognosis.

## Supplementary Information

**Additional file 1: Supplemental video 1.** Transthoracic echocardiography revealed strong echogenic foreign bodies in the ascending aorta and aortic arch, which were considered as guidewires.

**Additional file 2: Supplemental video 2.** The process of removing the guidewire in the aorta during sternotomy.

## Data Availability

The datasets of the current study are available from the corresponding author upon reasonable request.

## Abbreviations

CT: Computed tomography
DSA: Digital subtraction angiography
IAFB: Intra-aortic foreign bodies
